# Genome-WideAnalysis of Copy Number Variations in Three Populations of Nanyang Cattle Using Whole-Genome Resequencing

**DOI:** 10.3390/genes16050568

**Published:** 2025-05-12

**Authors:** Dong Dang, Lilian Zhang, Lutao Gao, Lin Peng, Yao Rao, Linnan Yang

**Affiliations:** 1 College of Big Data, Yunnan Agricultural University, Kunming 650201, China; 2 Yunnan Engineering Technology Research Center of Agricultural Big Data, Kunming 650201, China; 3 Yunnan Engineering Research Center for Big Data Intelligent Information Processing of Green Agricultural Products, Kunming 650201, China

**Keywords:** Nanyang cattle, copy number variation, genomic analysis, Vst

## Abstract

Copy number variation (CNV) serves as a crucial contributor to genetic diversity, exerting a profound influence on phenotypic diversity, traits of economic significance, and the evolutionary trajectory of livestock species. This study aimed to dissect the genome-wide CNV landscape of the Nanyang cattle line (Nanyang, Pinnan, and Xianan cattle) to identify functionally relevant CNVs associated with key economic traits and breed differentiation. In this study, 27 resequencing datasets were utilized to analyze the genome-wide distribution of CNVs in three breeds of Nanyang cattle (Nanyang cattle, Pinnan cattle, and Xianan cattle) based on the latest reference genome ARS-UCD2.0. This study identified a total of 97,564 CNVs, and after merging CNVs with overlapping genomic positions, we obtained 10,349 CNV regions (CNVRs), accounting for 1.48% of the reference genome. Functional enrichment analysis showed that CNVR genes were mainly involved in organ development, neural regulation, immune regulation, and metabolism. In addition, 131 CNVRs overlapped with 81 quantitative trait loci (QTLs), such as growth and carcass QTL, multiple birth QTL, tenderness score QTL, and antal follicle number QTL. Additionally, *AOX1, KRT72*, and *ZBTB7C* were found to overlap with body weight QTLs. Furthermore, a selective sweep analysis of CNVR revealed that numerous genes (*KIF26A, SPINT4, OR5W1*, etc.) exhibited divergent copy numbers between breeds. Conclusively, this study facilitates comprehension of the genetic characteristics of the Nanyang cattle line at the CNV level and furnishes valuable information for the advancement of the Nanyang cattle line breeding system.

## 1. Introduction

Copy number variations (CNVs) and single nucleotide polymorphisms (SNPs), as critical genomic variations, serve as key drivers in shaping domestication traits and adaptive evolution across animal and plant species [1]. Unlike SNPs, which refer to the substitution, deletion or insertion of only one nucleotide, CNV is defined as a change in the DNA sequence compared with the reference assembly due to the loss (deletion) or addition (insertion and duplication) of nucleotide bases. CNVs usually range from 1 kb to several Mb. Therefore, it is generally accepted that CNVs have the potential to significantly affect the phenotypic characteristics of livestock [2]. Consequently, enhancing our comprehension of the prevalence and functional intricacies of CNVs in livestock, particularly those associated with complex traits and environmental adaptation, will facilitate substantial advancements in the genetic enhancement of economic and production traits, along with animal health [3]. Previously, large-scale CNV detection was predominantly conducted using comparative genomic hybridization (aCGH) and high-density single-nucleotide polymorphism (SNP). However, these methods have certain limitations, including low coverage and low resolution. As sequencing costs decrease, next-generation sequencing (NGS) overcomes the limitations of chips and demonstrates significant advantages in the detection of genomic CNVs.

A substantial body of research has been dedicated to the analysis of CNV maps in various livestock species, including cattle, goats, sheep, and pigs. The findings of these studies have demonstrated that these CNVs have a considerable impact on the production performance of livestock. Studies have shown that CNVs were enriched in the immune system and olfactory receptor genes of cattle and were associated with some economic traits. The study also detected CNVs in the Kit gene, which is associated with color laterality [4]. Studies have found multiple CNV overlapping genes (such as EDNRA, ADAMTS20, and ASIP) related to adaptive traits (such as coat color, muscle development, and metabolic processes) [5]. Studies have found several important functional genes related to reproductive traits, such as *KDM2A, ACTN3, RHOD, ACTB, CCDC42, PIK3R5, NTN1,* and *BMP2*. These genes play a key role in embryonic development, spermatogenesis, cell proliferation, migration, and differentiation, and may affect the number of live piglets by changing gene dosage [6]. Studies have found that Lactalbumin Alpha (LALBA), a key gene that controls milk production in cattle, presents highly differentiated CNVs in the promoter region, making it a strong functional candidate gene for differences in milk production-related traits between swamp buffalo and river buffalo [7]. The duplication of RUNX Family Transcription Factor 1 (*RUNX1*) may promote hypoxia adaptation in OTS and HTS on the Qinghai–Tibet Plateau [8]. In addition, the distal-less homeobox 3 (*DLX3*) gene overlaps with the CNVR associated with wool curly, indicating that the CNV can be identified as a candidate for the special curly wool phenotype of Tan sheep [9].

Nanyang cattle stands as one of China’s five premier cattle breeds. During the agricultural epoch, this breed was indispensable, significantly contributing to the nation’s agricultural output. It holds a prominent place on the “National Inventory of Livestock and Poultry Genetic Resources”, as curated by China’s Ministry of Agriculture and Rural Affairs [10]. Prior research findings indicate that Nanyang cattle is a crossbreed resulting from the hybridization of Bos taurus and Bos indicus. This breed boasts notable strengths, including a towering build, superb meat texture, and a rich intramuscular fat content. However, it also exhibits certain drawbacks, such as a sluggish growth pace and a relatively low slaughter yield [11,12]. Pinnan cattle are a population formed by Piedmontese cattle as the father and Nanyang cattle as the mother through progressive hybridization, cross-breeding, and self-breeding. This results in an early puberty, a fast growth rate, and an excellent meat quality [13]. Xianan cattle are a novel breed formed by Charolais cattle as the father and Nanyang cattle as the mother through cross-breeding innovation and the backcrossing of superior individuals. Xianan cattle exhibit several advantageous traits, including early maturity, accelerated growth, superior meat quality, and a reduced incidence of dystocia, thereby enhancing the efficiency of beef cattle production and the economic returns to the cattle industry [14]. Up until now, research efforts in this field have been rather sparse, with only a handful of studies delving into the genomic disparities within the Nanyang cattle breed at the levels of single nucleotide polymorphisms (SNPs) and insertions/deletions (INDELs). Moreover, the exploration of copy number variation (CNV) in this breed has predominantly centered around the influence of CNV in a solitary gene on growth-related traits, leaving broader genomic patterns and interactions largely uncharted [10,15].

This study aimed to characterize genome-wide copy number variation patterns across three Nanyang cattle lineages (Nanyang, Pinnan, and Xianan cattle) using high-coverage resequencing data, with the following objectives: (1) systematically map CNVRs and assess their genomic distribution and functional relevance to key economic traits; (2) identify CNVRs overlapping quantitative trait loci (QTLs) associated with growth, carcass quality, and reproductive performance; and (3) investigate population-level differentiation in CNVRs through selective sweep analysis to uncover signatures of artificial or environmental selection. By integrating comparative genomics, functional annotation, and population genetics approaches, this work seeks to establish a foundational CNV resource for the Nanyang cattle line, enabling targeted exploration of structural variation in breeding programs and advancing molecular strategies for trait optimization.

## 2. Materials and Methods

### 2.1. Samples Collection and Genome Sequencing

In this study, a total of 27 whole genome sequencing data sets of cattle samples were obtained, all of which were downloaded from the NCBI public database. The data sets included 7 Nanyang cattle (https://www.ncbi.nlm.nih.gov/bioproject/?term=PRJNA396672, https://www.ncbi.nlm.nih.gov/bioproject/?term=PRJNA379859, accessed on 16 March 2025), 10 Pinnan cattle (https://www.ncbi.nlm.nih.gov/bioproject/?term=PRJNA698276, accessed on 16 March 2025) and, 10 Xianan cattle (https://www.ncbi.nlm.nih.gov/bioproject/?term=PRJNA1058368, accessed on 16 March 2025) (Supplementary Table S1). All data were sequenced on an Illumina HiSeq 2000 sequencer (Illumina Inc., San Diego, CA, USA) with 100 bp paired-end reads. Following the acquisition of the downloaded cattle whole genome sequencing raw data, the raw data of the Illumina platform were filtered using FASTP software version 0.18.0 [16] to obtain relatively high-quality sequencing data for clean read assembly analysis. The raw reads were processed according to stringent filtering criteria: (1) removal of reads with ≥10% unidentified nucleotides (Ns), (2) removal of reads where >50% of bases had a Phred quality score ≤ 20, and (3) removal of reads aligned with adapter sequences [17]. The preprocessed sequencing data underwent alignment against the most recent cattle reference genome, ARS-UCD2.0, which was sourced from the Ensembl database. This alignment was executed utilizing the BWA software package [18], and Subsequently, to ensure the accuracy of CNV analysis, any PCR duplicates that could potentially skew the results were systematically identified and eliminated using the Picard-2.9.2 (https://broadinstitute.github.io/picard/, accessed on 16 March 2025) and its Markduplicates module.

### 2.2. Detection of CNVs and CNVRs

We detected CNVs for each individual by using CNVnator [19], Lumpy [20], and CNVcaller [21]. We then merged the results of CNVnator and Lumpy with the results of CNVcaller, with the goal of maximizing population-specific variation while reducing rare variation at the individual level. CNVnator operates as a read-depth analysis tool tailored for detecting CNV by comparing genomic data against the ARS-UCD2.0 reference genome. To enhance the precision of CNV predictions, stringent filtering criteria were applied: calls were retained only if they met a *p*-value threshold of less than 0.001, had a proportion of reads with zero mapping quality (q0) below 0.5, and spanned a genomic region exceeding 1 kilobase in size. For the purpose of cross-variety comparisons in copy number, the “-genotype” feature within CNVnator was employed to derive an estimated copy number count for each genomic segment of interest. The Lumpy software, configured with its default parameter settings, was utilized to identify copy number variations (CNVs). Specifically, for each sample analyzed, the Lumpy express module was engaged to scrutinize discordant-read pairs and split-read pairs, processes which are instrumental in accurately detecting and delineating genomic regions exhibiting copy number alterations. We used manual inspection and SURVIVOR (version 0.0.1) [22] to merge the results of the three software and determine the final data set. The CNVRs of the Nanyang cattle line were divided into duplication CNVRs, deletion CNVRs, and CNVRs with both duplication and deletion. The length of the CNVRs with both deletion and duplication and deletion types did not exceed 50 kb, and the length of the CNVRs with duplication types did not exceed 500 kb [23]. Furthermore, an analysis was conducted to examine the chromosomal distribution patterns of these specific genomic regions within the Nanyang cattle lineage. This was achieved by utilizing the RIdeogram tool [24], which is part of the Bioconductor software suite.

### 2.3. Functional Annotation and Enrichment Analysis of CNVRs

To decode the functional implications of the detected CNV within the Nanyang cattle breed’s genetic blueprint, we retrieved the corresponding annotation files from the NCBI database, specifically from the directory (https://ftp.ncbi.nlm.nih.gov/genomes/all/GCF/002/263/795/GCF_002263795.3_ARS-UCD2.0/, accessed on 16 March 2025). These annotations offer a detailed map of the genomic landscape, enabling us to explore how the CNVs may shape the breed’s phenotypic traits and biological processes. The annotation of candidate CNVRs was completed by using the software program, ANNOVAR [25]. We conducted Gene Ontology (GO) enrichment analysis and Kyoto Encyclopedia of Genes and Genomes (KEGG) pathway analysis, focusing solely on protein-coding genes. This analytical process was carried out utilizing the Database for Annotation, Visualization, and Integrated Discovery (DAVID), accessible via the URL (https://david.ncifcrf.gov/, accessed on 16 March 2025). The aim was to uncover the biological processes, molecular functions, and cellular components associated with the genes of interest, as well as to identify the relevant signaling pathways and metabolic networks they might be involved in [26]. Biological process, cellular component, and molecular function were used as GO term categories with a significance level of 0.01. Furthermore, quantitative trait loci (QTLs) for cattle were downloaded from the cattle QTLdb (https://www.animalgenome.org/cgi-bin/QTLdb/BT/summary, accessed on 16 March 2025) [27] and compared with the identified CNVRs. Given the dearth of research focusing on relevant quantitative trait loci (QTLs) mapped against the ARS-UCD2.0 genome assembly, our investigation was constrained to utilizing QTL data documented in the ARS-UCD1.2 version. Specifically, we filtered for QTLs with a confidence interval narrower than 5 megabases (Mb), aiming to refine our analysis to regions of higher genomic precision. To ascertain the overlap between these QTLs and the CNVRs detected in our study, we leveraged the ’intersect’ functionality of the Bedtools-v2.27.1 software package [28]. This approach enabled us to pinpoint QTLs that spatially coincide with CNVRs, thereby offering insights into potential genetic loci influencing traits of interest in Nanyang cattle.

### 2.4. Sweep Selective Analysis of the CNVR

To pinpoint copy number variation regions (CNVRs) that differ among Nanyang, Xianan, and Pinnan cattle breeds, we computed the Vst statistic. Vst operates on principles akin to the Fst statistic, a well-established measure for assessing genetic differentiation between populations. However, Vst is tailored to quantify population disparities based on copy number data.The formula for calculating Vst is expressed as Vst = (Vt − Vs)/Vt. In this equation, Vt denotes the total variance observed across all unrelated individuals from the combined cattle populations. On the other hand, Vs signifies the weighted average of the variances within each individual population, with the weights assigned according to the respective population sizes [29]. Subsequently, we zeroed in on the upper 5% of CNVR that exhibited exceptionally elevated Vst values. These extreme-value loci, effectively serving as “outliers” within our dataset, were scrutinized to investigate their potential links with prominent phenotypic traits specific to Nanyang cattle. To unravel the underlying biological mechanisms and functional roles these regions might play, we conducted a comprehensive functional enrichment analysis on these selected CNVRs.

## 3. Results

### 3.1. The Landscape of Copy Number Variation in Nanyang Cattle

**Figure 1:**
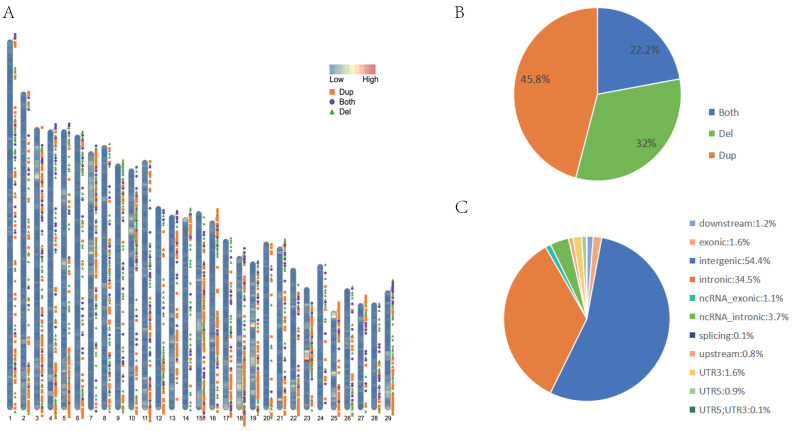
Genetic diversity and distribution of CNVRs in Nanyang cattle. (**A**) Autosomal distribution of CNVRs. The colors painted on the chromosomes represent gene density, and the positions of different colors outside the chromosomes represent duplications (orange), deletions (green), and duplications and deletions (purple). (**B**) The frequency of different types of CNVRs. (**C**) Functional classification of the detected CNVRs.

We gathered whole genome sequencing data from a cohort of 27 Chinese cattle breeds. The sequencing depth for these samples ranged between approximately 5.43X and 13.91X. Once the sequencing reads were generated, we aligned them to the bovine reference genome, ARS-UCD2.0. This alignment step is crucial as it allows us to accurately map the genetic information from our samples onto a standardized genomic framework. Following the alignment process, we achieved an impressive average coverage rate of 99.68%. Such a high level of coverage is vital as it significantly bolsters the reliability of our subsequent CNV detection efforts, ensuring that the genetic variations we identify are based on a robust and comprehensive genomic dataset (Supplementary Table S1). We generated a CNVR dataset for each cattle breed, which contained 10,349 CNVRs, including 4741 duplicate CNVRs, 3313 deleted CNVRs, and 2295 duplicated and deleted CNVRs, with a total length of 36,814,051 bp and an average length of 3557 bp, covering 1.48% of the reference genome (Figure 1A). In this study, 10,349 CNVRs (comprising duplications, deletions, and duplications and deletions) were categorized into groups of varying lengths. The size distribution of all CNVRs exhibited an L-shaped curve, with 50.9% of CNVRs situated within the 0–2 kb range, 30.7% of CNVRs located within the 2–5 kb range, 6.8% of CNVRs positioned within the 5–10 kb range, and the remaining CNVRs exceeding 10 kb (Figure 1B) (Supplementary Table S2). Upon closer examination, it became evident that the distribution of CNVR across the genome was far from uniform. A substantial majority, accounting for 54.3% (or 5621 in number) of the CNVRs, were situated within intergenic regions—those expanses of DNA lying between genes. In stark contrast, only a minuscule 1.6% of the CNVRs were found nestled within exonic regions, which are the protein-coding segments of genes crucial for determining an organism’s traits (Figure 1C).

### 3.2. Functional Annotation of CNVRs

Functional enrichment analysis was performed on GO terms and KEGG pathways throughDAVID with 2851 protein-coding genes within 1 kb of CNVR [30]. The 10,349 identified copy number variation regions (CNVRs) were mapped to a curated set of 2851 genes, revealing a subset of the genome where structural alterations in copy number have occurred. These genes, which are prone to undergoing copy number variations, represent an exceptionally precious genomic asset. They offer a unique avenue for delving into the complex web of connections that exist between genes affected by CNV and the observable physical and behavioral characteristics in Nanyang cattle. To gain a more profound insight into how these CNVR functionally impact biological processes, we performed an extensive functional enrichment analysis focusing on the genes located within these specific genomic areas. The DAVID database was utilized to perform functional enrichment analysis of GO terms and KEGG pathways on the 2851 genes. The analysis identified 173 GO terms that were significantly enriched (*p* < 0.01), encompassing 76 biological processes, 54 cellular components, and 43 molecular functions (Supplementary Table S3). The functions of these genes are primarily associated with organ development, neural regulation, immune regulation, and metabolism. Examples of specific terms include protein binding (GO:0005515), ATP binding (GO:0005524), metal ion binding (GO:0046872), and signal transduction (GO:0007165). Furthermore, a KEGG pathway analysis of shared CNVR hidden genes revealed their enrichment in 48 pathways (Supplementary Table S4, *p* < 0.05), including the calcium signaling pathway (bta04020), the Focal adhesion pathway (bta04510), the Oxytocin signaling pathway (bta04921), and the cell adhesion molecules pathway (bta04514), amongst others.

### 3.3. QTLs Overlapping with Identified CNVRs

To further elucidate the correlation between CNVRs and traits in Nanyang cattle, we used QTL data from cattle for comparison with the detected CNVRs. The results of this analysis indicated that 131 CNVRs were found to overlap with 81 quantitative trait loci (QTLs), including Subcutaneous fat thickness QTL (38 CNVRs), Longissimus muscle area QTL (14 CNVRs), multiple birth QTL (14 CNVRs), tenderness score QTL (11 CNVRs), Antral follicle number QTL (9 CNVRs), etc. (Supplementary Table S5). Furthermore, we identified several CNVR genes associated with slaughter performance, including *AOX1, KRT72, SFXN1, ZBTB7C*, and *CACNA1G* genes located at Longissimus muscle area QTL (223729), Meat color QTL (222285), Marbling score QTL (222248), and multiple birth QTL (258520). These data hold immense significance in guiding future genetic enhancement efforts aimed at advancing the Nanyang cattle breed.

### 3.4. CNVRs Diverging Among Populations

**Figure 2:**
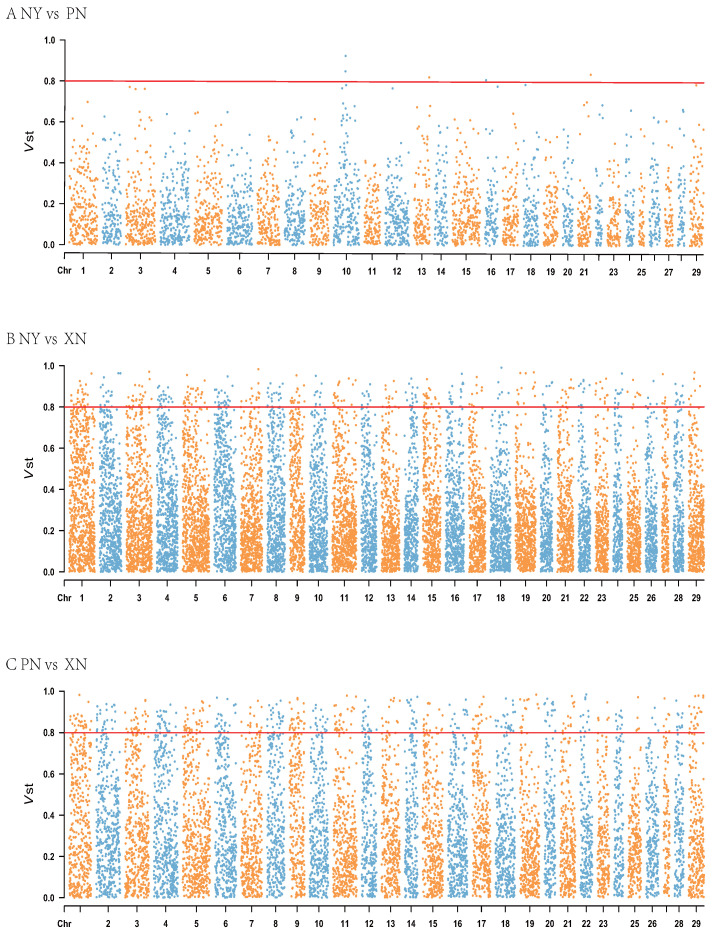
The Manhattan plot of the gene Vst values of the whole genome. (**A**) NY vs. PN. (**B**) NY vs. XN. (**C**) PN vs. XN. The red line indicates 0.8 Vst.

We applied Vst statistics to analyze the differentiation of CNVR among Nanyang cattle, Pinnan cattle, and Xianan cattle. The average Vst values of all detected responses to CNVR were 0.1591 for Nanyang cattle and Pinnan cattle, 0.2561 for Nanyang cattle and Xia’nan cattle, and 0.3157 for Pinnan cattle and Xianan cattle (Supplementary Table S6). Pinnan and Xianan cattle showed the highest degree of differentiation, which is consistent with the results of breeding between the two cattle. As shown in Figure 2 and Supplementary Table S6, different CNVRs were unevenly distributed on chromosomes. To understand the genes with high differentiation between breeds, we further examined genes with VST > 0.79 (the highest 98th percentile). Four genes including *LOC788997, KIF26A, SPINT4*, and *OR5W1* in Nanyang cattle and Pinnan cattle, 407 genes including *CEBPA, LOC101905257, TWIST2, KCNJ5,* and *CRLF3* in Nanyang cattle and Xianan cattle, and 529 genes including *IFRD2, SLC16A5, LOC101905099, LRP5*, and *CLMN* in Pinnan cattle and Xianan cattle all exceeded the threshold. Further functional analysis showed that a total of 14 GO terms were found to be enriched between Nanyang cattle and Xianan cattle, which were mainly related to neural regulation and control. In addition, 16 KEGG pathways were enriched, including Dopaminergic synapse, Oxytocin signaling pathway, and calcium signaling pathway (Supplementary Table S7). A total of 23 GO terms were found to be enriched between Pinnan cattle and Xianan cattle, which were mainly related to signal transduction and organ development. In addition, 20 KEGG pathways were enriched, including Circadian entrainment, Cortisol synthesis and secretion, and Regulation of actin cytoskeleton (Supplementary Table S8).

## 4. Discussion

Throughout the processes of domestication and subsequent diversification within a species, the prevalence of CNV in its genome dynamically shifts in response to selective pressures exerted by environmental demands or human-driven breeding goals, while substantial research endeavors have focused on pinpointing causal mutations and pivotal genes underlying traits of interest, the task of systematically screening and validating genomic markers linked to copy number changes remains inherently challenging due to their structural complexity and the intricate interplay of genetic factors. As a prominent source of genetic diversity distinguishing individuals within a population, CNVs hold significant potential to drive phenotypic alterations. They can exert their influence through multiple mechanisms, such as disrupting gene architecture, altering gene dosage (thereby modifying the number of gene products), and perturbing the delicate balance of allele frequencies that govern regulatory networks. These effects underscore the critical role of CNVs in shaping the genetic and phenotypic landscape of a species, highlighting their importance in evolutionary trajectories and breeding strategies [31]. Over the past several decades, the advent and rapid advancement of high-throughput sequencing (HTS) methodologies, coupled with sophisticated bioinformatics analytics, have progressively revolutionized the field of genomic research. These technological innovations have been pivotal in enabling the construction of comprehensive, genome-wide maps of CNV, offering unprecedented resolution and scale in the study of structural genomic diversity [32]. The diversity of CNVs has been extensively explored in various animals, such as cattle, sheep, chickens, and pigs.

In our present investigation, we leveraged whole genome sequencing data generated through Next Generation Sequencing (NGS) technology to uncover CNV. When contrasted with conventional CNV-detection approaches, such as those relying on SNP microarrays and array Comparative Genomic Hybridization (aCGH), NGS offers a host of benefits in accurately quantifying both the quantity and dimensions of CNVs. Thanks to its exceptional sensitivity in CNV detection, NGS is capable of pinpointing CNV boundaries with a far greater degree of precision [33]. Compared with the ARS-UCD1.2 reference genome, it improves the reliability of CNV screening. To accurately gauge copy numbers at genomic breakpoints and structural variation hotspots, we employed three distinct software tools, each employing unique algorithms tailored for precise CNV detection. Our results showed that copy number duplication events were more common than deletion events, which is consistent with most previous reports. In addition, the location distribution of CNVRs in the cattle genome is not uniform and is non-randomly scattered on chromosomes. Genomic annotation revealed that a substantial proportion of CNVR was predominantly mapped to intergenic or intronic segments within the cattle genome (Figure 1C). This finding aligns with prior research, which similarly indicates that numerous CNVRs are situated within genomic loci characterized by high variability, often encompassing genes with dynamic regulatory or structural features [23].

In the present study, GO enrichment analysis showed that many CNVR-carrying genes were significantly enriched with GO terms related to sensory perception (Supplementary Table S3). This is consistent with findings from studies of CNVs in humans, yak, pigs, horses, dogs, and mice, which also found significant enrichment of GO terms related to sensory perception [34,35,36]. In addition, GO terms related to energy metabolism were also significantly enriched. Fine regulation of energy metabolism plays a decisive role in the healthy growth and reproduction of cattle under different climate conditions and food resource supplies. Through KEGG signaling pathway analysis, it was found that CNVR genes were significantly enriched in signal transduction and nutritional metabolism (Supplementary Table S4). The calcium ion signaling pathway represents a pivotal signaling mechanism within cells, exhibiting significance across a diverse array of cell types and functions. It has been demonstrated to regulate a number of processes, including cell proliferation, differentiation, apoptosis, and various physiological functions. This is achieved by regulating changes in the concentration of calcium ions within the cell, transmitting information and triggering a series of biological reactions. Furthermore, the enrichment of CNVR overlapping genes on nervous system-related signaling pathways in Nanyang cattle breeds has established a genetic foundation that enables adaptation to diverse environmental challenges. These gene enrichments enable the Nanyang cattle line to respond more effectively to various environmental changes by optimizing neural responses and behavioral patterns. This discovery does far more than simply enrich our comprehension of how cattle have evolved adaptively over time; it also paves the way for innovative avenues and fresh conceptual frameworks in future research endeavors. These will be focused on enhancing the genetic traits of Nanyang cattle breeds and refining their capacity to thrive in diverse environmental conditions.

The QTL analysis in this study showed that many CNVR overlapping genes were located in the growth and carcass QTL regions, such as *AOX1, KRT72, SFXN1, ZBTB7C*, etc. The levels of 2-pyrrolidone and glycerophospholipids are regulated by the gene expression of *AOX1*, which further affects the levels of volatiles, 2-pyrrolidone, and decanal, respectively [37]. The *KRT7* gene exhibited elevated expression levels within the phenotype group, suggesting that keratin proteins play a role in the manifestation of the plaque-associated phenotype. Keratin proteins, collectively denoted as *KRTs*, constitute the primary structural constituents of skin, hair, and wool. They exert regulatory influence over the growth and developmental processes of these tissues [38]. *Sfxn1* is essential for erythrocyte maturation via facilitating hemoglobin production in zebrafish [39]. The *ZBTB7C* gene functions as a regulatory hub, governing the expression of Matrix Metalloproteinases (MMPs)—a family of zinc-dependent endopeptidases with multifaceted roles in cellular processes. MMPs are pivotal in orchestrating cell proliferation, migration, and differentiation, as well as in regulating angiogenesis and apoptosis [40]. Given these critical functions, the genes harboring CNV identified in this study emerge as promising molecular markers. These markers hold significant potential for guiding future breeding strategies aimed at enhancing the Nanyang cattle lineage, offering a genetic basis for targeted improvements in traits relevant to livestock production and health.

Selective sweep analysis serves as a powerful tool to uncover critical genomic regions that harbor candidate genes shaped by both environmental pressures and artificial selection throughout the processes of adaptation and domestication [41]. Notably, in our study, the unconventional kinesin *KIF26A*. This gene exerts a pivotal influence on the development of the enteric nervous system (ENS), functioning by suppressing a cell growth-promoting signaling cascade [42]. *SPINT4* is an epididymis-specific protein having anti-proteolytic activity, which is related to spermatozoal maturation, motility, and male fertility [43]. Functional enrichment assessments revealed that genes co-localized within the distinct CNVRs were predominantly implicated in vital biological pathways. These pathways encompassed regulatory mechanisms and the calcium signaling cascade, shedding light on their substantial contribution to the adaptive disparities observed across different cattle breeds.

While this genome-wide CNV profiling of Nanyang cattle populations provides valuable structural variation data, two validation layers remain outstanding. Firstly, the absence of orthogonal biological validation (qPCR or droplet digital PCR confirmation for high-impact CNVRs) introduces uncertainty in boundary delineation accuracy, particularly for complex tandem duplications. Secondly, the phenotypic correlative analysis is constrained by unavailable quantitative trait datasets, could not be integrated through mixed linear models (GWAS-based CNV–phenotype associations). Future investigations should prioritize mediated CNV reconstruction in bovine primary myocytes coupled with transcriptomic profiling to empirically verify dosage effects on candidate genes.

## 5. Conclusions

The Nanyang cattle lineage exhibits lineage-specific CNVRs enriched in calcium signaling, cell adhesion, and Oxytocin pathways, directly linking structural genomic variation to enhanced muscle development, tissue integrity, and reproductive efficiency. Overlap analysis identified 131 CNVRs colocalized with 81 QTLs, including *AOX1* (associated with meat flavor compounds) and *SFXN1*(linked to meat tenderness), implicating copy number dosage effects in carcass quality optimization. Selective sweep analysis revealed divergent CNVRs (*KIF26A* and *SPINT4*) under breed-specific selection, reflecting adaptive pressures on neuronal regulation and reproductive traits. Notably, 54.4% of CNVRs reside in intergenic regions, suggesting regulatory element disruption as a driver of phenotypic diversity. Breed-specific copy number differences in *OR5W1* (olfaction) and *LRP5* (bone density) further highlight genetic adaptations to ecological niches and management practices, providing actionable insights for precision breeding programs targeting productivity and resilience.

## Data Availability

The datasets presented in this study can be found in online repositories. The names of the repository/repositories and accession number(s) can be found below: https://www.ncbi.nlm.nih.gov/, PRJNA379859, PRJNA396672, PRJNA698276, PRJNA1058368.

## Supplementary Materials

The following supporting information can be downloaded at: https://www.mdpi.com/article/10.3390/genes16050568/s1, Tables S1–S8.
